# University Students’ Views on the Perceived Benefits and Drawbacks of Seeking Help for Mental Health Problems on the Internet: A Qualitative Study

**DOI:** 10.2196/humanfactors.4765

**Published:** 2016-01-19

**Authors:** Jade KY Chan, Louise M Farrer, Amelia Gulliver, Kylie Bennett, Kathleen M Griffiths

**Affiliations:** ^1^School of PsychologyFaculty of ScienceUniversity of New South WalesSydneyAustralia; ^2^National Institute for Mental Health ResearchThe Australian National UniversityCanberraAustralia; ^3^Young and Well CRCMelbourneAustralia

**Keywords:** online, mental health, help seeking, university, students, qualitative

## Abstract

**Background:**

University students experience high levels of mental health problems yet very few seek professional help. Web-based mental health interventions may be useful for the university student population. However, there are few published qualitative studies that have examined the perceived benefits and drawbacks of seeking help for mental health problems on the Internet from the perspective of university students.

**Objective:**

To investigate the attitudes of university students on mental health help-seeking on the Internet.

**Methods:**

A total of 19 university students aged 19-24 years participated in 1 of 4 focus groups to examine their views toward help-seeking for mental health problems on the Internet.

**Results:**

Perceived concerns about Web-based help-seeking included privacy and confidentiality, difficulty communicating on the Internet, and the quality of Web-based resources. Potential benefits included anonymity/avoidance of stigma, and accessibility. Participants reported mixed views regarding the ability of people with similar mental health issues to interact on the Internet.

**Conclusions:**

These factors should be considered in the development of Web-based mental health resources to increase acceptability and engagement from university students.

## Introduction

Approximately 30% of university students meet criteria for a mental disorder [1,2]. The most commonly reported disorders in this population are substance use disorders, personality disorders, depression, anxiety disorders, and eating disorders [3]. Risk factors for developing a mental disorder among university students include experience of a stressful life event, living away from home, and experience of financial stress [2,3]. Despite the severity of the negative outcomes associated with untreated mental disorders, including poorer interpersonal relationships, lower engagement in campus activities, and increased risk of educational dropout [4], very few students receive appropriate mental health care [1]. Given the barriers to seeking treatment reported by students (eg, fear of stigma, cost, and time constraints) [5,6], Web-based mental health interventions, such as peer-to-peer forums, screening tools, and educational and self-help programs, may be highly suited to the university population. Web-based interventions are easily accessible, can be utilized in private, are cost-effective, and typically require less time than face-to-face appointments [7]. Moreover, there is evidence that Web-based interventions targeting mental health are effective for university students [8] and that university students hold favorable attitudes towards Web-based mental health resources [9]. However, there is little exploration of concerns that university students may have regarding seeking help on the Internet, or why they may find Web-based mental health resources appealing. Qualitative research allows in-depth exploration of students’ perceptions of these issues, which has key implications for the design of Web-based mental health resources targeting this population. The current study aims to investigate the attitudes of university students towards seeking mental health support on the Internet.

## Methods

### Design

A total of 4 focus groups were conducted with students from a large university in Canberra, Australia. Each focus group consisted of 4 or 5 participants (*n*=5, 5, 4, 5) with a total of 19 participants. Focus groups were chosen due to their potential to generate in-depth discussion. Given the general nature of the questions asked, discussion of personal experiences was not required. The duration of each session was approximately 1.5 hours. Ethics approval was granted by The Australian National University (ANU) Human Research Ethics Committee (2012/520). Cinema vouchers were offered to participants for their time and involvement in the focus groups.

### Participants

Of the 19 university students who participated in the focus groups, 10 were women. The mean age of the sample was 21.6 years (range 19-24 years) and the mean duration of their university education was 3.1 years (range 1-5 years). Most participants were domestic students (*n*=12, 63%) from a range of disciplines (arts, law, commerce, engineering, science, music, and combined degrees from those faculties). Participants were recruited via email invitations distributed to students in residential colleges and students who had previously expressed interest in participating in mental health research.

### Procedure

All focus groups were conducted by the primary facilitator (LF), a female postdoctoral research fellow and registered psychologist at the National Institute for Mental Health Research (NIMHR). During focus group meetings, 2 research assistants (AG and JC) were present to provide assistance and take field notes. The focus groups were audio recorded. On arrival, participants were provided with an information sheet, a consent form, and a short demographic questionnaire. At the beginning of each focus group session, the primary facilitator provided a brief introduction to the study and information about the purpose of the focus groups, confidentiality, and voluntary participation. Focus group participants were asked a series of questions relating to help-seeking preferences for mental health problems (both offline and on the Internet) and their views toward the development of a virtual mental health clinic for university students. Data relevant to the development of the virtual clinic have been published elsewhere (see [10] and [11]). The current paper focuses on participant responses to the following question: *“What do you think about using the Internet to get support for mental health or emotional problems?”* Participants who sought clarification about the meaning of “using the Internet” were provided with typical examples of Web-based mental health resources (eg, informational websites, self-help therapy programs, and peer-to-peer support networks such as forums and chat platforms).

### Analysis Strategy

Data were analyzed using thematic analysis in NVivo 10 by the first author (JC). Participants’ statements in response to the question above were coded based on a grounded theory approach [12], whereby similar concepts were grouped together into themes. During the discussion of Web-based help-seeking 2 major themes emerged: “concerns” and “benefits.” Direct quotes are used to illustrate the themes and participants are identified by gender and a coded number (eg, F1 = female, participant number 1). The number of participants who endorsed each benefit and concern are also reported.

## Results

### Concerns Regarding Web-Based Mental Health Support

Participants reported a number of concerns about seeking help for mental health problems on the Internet including privacy and confidentiality (n=2), resource quality (n=3), and communication difficulties (n=4). A major concern was privacy and confidentiality:

I’d be worried about putting things in writing in the Internet. I don’t know why. Like I know...that there’ll have to be confidentiality around it, but the Internet just seems kind of insecure.

F1

Participants noted that while there is a wealth of mental health information on the Internet, it is scattered and difficult to find, which was viewed as a barrier to seeking help:

There’s just so much, and that it’s not really centralized and, you know, it’s about knowing where to go as well.

F2

There was also skepticism about the quality and accuracy of information available on the Internet, as well as the ability for Web-based resources to address individualized problems:

I probably think it’s a bit too generic for like, a personal problem.

F3

A final concern that emerged was that participants felt that it could be difficult to accurately portray emotions through writing on the Internet:

I think talking and typing it out is also different...It is a good idea but...maybe someone just can’t get that across to someone else when you’re just typing it out.

F4

### Benefits of Seeking Mental Health Support on the Internet

Participants reported several benefits of using Web-based resources to address mental health problems. These included anonymity/avoidance of stigma (n=3) and accessibility (n=3). The ability to seek help without other people knowing (and potentially passing judgement) was seen as particularly important:

The Internet is very useful and it’s almost an entirely anonymous way to gather information so you can use it without, sort of revealing anything about yourself, about you personally...you can just take information without having to give any.

M2

Accessibility after business hours was also raised as an advantage of Web-based resources given that, in the participants’ experience, mental health crises tended to occur at night.

It’s readily accessible, so...if you’re out of hours obviously you can go and see, like on a first-hand basis what...some advice is.

F3

### Mixed Views

During a discussion of Web-based forums, participants reported mixed views regarding the ability for people with past or current experience of similar mental health problems to seek support from one another on the Internet. One participant expressed concern (n=1) that Web-based forums may compromise safety by enabling interaction between people who are distressed, thereby exacerbating their problems.

If you’ve got a bunch of really depressed people all together in a kind of, I guess, confined Internet space, they just make each other more depressed.

M1

For others (n=2), the ability of the Internet to connect people experiencing similar issues (eg, via forums) was viewed very positively:

I definitely feel it could be an important tool...I think there’s no better way to help someone out than someone who’s actually been through the same problems as you have.

M3

## Discussion

### Principal Findings

When asked directly about their views toward seeking Web-based support for mental health problems, participants raised a number of concerns. These related primarily to the privacy of Web-based resources, which has been noted as a key issue to consider in the development of health websites [13-16]. Additionally, the difficulty of communicating effectively on the Internet was raised. This echoes previous findings in a sample of university students [17], where students who preferred face-to-face therapy cited facilitation of communication as a primary reason for their preference. Additionally, difficulty with communication has previously been raised as a potential issue in Internet therapy [18]. However, Abbott and colleagues suggested this problem could be minimized by the user seeking clarification from the message source if there is a concern or misunderstanding [18].

Several participants mentioned the desire for centralized information and resources, suggesting that young people may feel overwhelmed when consulting the Internet for help with their mental health. Developers of Web-based mental health websites or interventions should aim to consolidate and present resources in a navigable and accessible way. Increasing awareness of websites with centralized information may also be beneficial to university students.

However, several positive attitudes toward Web-based help-seeking were raised, including the ability to access help privately and after hours. These are often cited as advantages of Web-based resources (eg, [7]). The Internet was also viewed as filling a gap for students who may wish to remain anonymous or avoid stigma, which is consistent with previous studies with other groups of young people [19]. The participants in the current study endorsed the idea of a Web-based forum for young people to relate to one another. Significantly, prior research has demonstrated that more than half of young people aged 18-25 years use forums for connecting with their peers, and for talking about their problems on the Internet [20]. However, moderation of Web-based interaction in forums is an important consideration for the development of a university virtual clinic, given the concern raised that forum discussion could exacerbate psychological distress. Participants also expressed a desire for relevant, centralized resources. Overall, a virtual clinic that offers tailored information, self-help programs, access to professional support, confidential screening and feedback, and peer support tools is likely to appeal to this population.

### Limitations

There are several limitations to the present research that require consideration. Participants were not selected on the basis of current or previous experience with a mental health problem, and their mental health status was not assessed. It is unclear to what extent the views and intentions of students without mental health problems are applicable to those of students with a mental illness. However, several students chose to disclose either personal or close family members’/friends’ experiences of a mental health problem. The views of all university students were considered valid for the purposes of the study, given that the aim of the virtual university clinic is to provide prevention services as well as treatment. Because participants self-selected to participate and this study was conducted in one university setting, the views expressed may not represent the views of all students within the participating university more broadly, or students from other universities. Finally, findings in the current paper are limited to the responses to a specific question of interest.

### Implications

The findings of this research have implications for the development of Web-based mental health resources for university students. Despite their potential impact during emerging adulthood, mental health problems still remain undertreated in this vulnerable group, in part due to barriers to help-seeking (eg, cost, time, accessibility). Web-based mental health resources have the potential to overcome these barriers; however, in order to optimize acceptability and engagement, it is critical that the concerns of university students are taken into account in the development of these resources in universities. Web-based resources should seek to offer security of information, anonymity, and treatment that is tailored to an individual’s needs. Involving students in the intervention development process as co-designers may also address some of their concerns and improve eventual uptake of these services.
